# Study of the effect of higher-order dispersions on photoionisation induced by ultrafast laser pulses applying a classical theoretical method

**DOI:** 10.1038/s41598-022-18034-w

**Published:** 2022-08-16

**Authors:** István Márton, László Sarkadi

**Affiliations:** 1 MTA Atomki Lendület Quantum Correlations Research Group, Institute for Nuclear Research, P.O. Box 51, Debrecen, H-4001 Hungary; 2 Institute for Nuclear Research, (Atomki), P.O. Box 51, Debrecen, H-4001 Hungary

**Keywords:** Atomic and molecular interactions with photons, Ultrafast photonics

## Abstract

We investigated the effect of higher order dispersion on ultrafast photoionisation with Classical Trajectory Monte Carlo (CTMC) method for hydrogen and krypton atoms. In our calculations we used linearly polarised ultrashort 7 fs laser pulses, $6.5 \times 10^{14} \mathrm {W/cm^{2}}$ intensity, and a central wavelength of 800 nm. Our results show that electrons with the highest kinetic energies are obtained with transform limited (TL) pulses. The shaping of the pulses with negative second- third- or fourth- order dispersion results in higher ionisation yield and electron energies compared to pulses shaped with positive dispersion values. We have also investigated how the Carrier Envelope Phase (CEP) dependence of the ionisation is infuenced by dispersion. We calculated the left-right asymmetry as a function of energy and CEP for sodium atoms employing pulses of 4.5 fs, 800 nm central wavelength, and $4 \times 10^{12}\mathrm {W/cm^{2}}$ intensity. We found that the left-right asymmetry is more pronounced for pulses shaped with positive Group Delay Dispersion (GDD). It was also found that shaping a pulse with increasing amounts of GDD in absolute value blurs the CEP dependence, which is attributed to the increasing number of optical cycles.

## Introduction

Dispersion plays an important role in ultrafast optics^1^. The chirped pulse amplification^2^ technique is based on the proper control of dispersion that made possible the generation of ultrafast laser pulses with petawatt peak power^3^. Dispersion also has fundamental effect in chemical processes^4^. Numerous studies show the second-order dispersion, also called Group Delay Dispersion (GDD) dependence on photoelectron spectra^5,6^ or photoionisation yield^7^ from molecules. Reusch et al.^7^ demonstrated a higher photoelectron yield from hydrocarbon molecules with negatively chirped pulses. Another group investigated the photodissociation process of the $\mathrm {H_2^+}$ molecule as a function of GDD^8^ and Third Order Dispersion (TOD)^9^. The investigations indicated that dissociation of the molecule was more probable with pulses shaped with positive GDD and negative TOD compared to pulses with negative GDD and positive TOD. Second- and third-order dispersions were also used to distinguish the isomers of complex organic molecules^10,11^. Theoretical investigations predicted that the spectra and angular distribution of Above-Threshold Ionisation (ATI) by ultrashort laser pulses are influenced by the chirp parameter of the ultrashort pulse^12^ for Na atom. Theoretical and experimental investigations show the influence of the chirp parameter on the photoelectron spectra from sodium^13^ and potassium atoms^14^. It was verified that the photocurrent from planar surfaces induced by a few-cycle pulse depends on the Carrier Envelope Phase (CEP)^15^. The CEP dependence is manifested by an asymmetry along the polarization axis of the incoming pulse for both nanoparticles^16^ and atoms^17,18^. Namely, the number of electrons emitted with negative and positive momentum are not the same, known as left-right asymmetry. Recently it was verified for sodium atoms both experimentally and theoretically that this asymmetry is influenced by the chirp of the pulse^19^.

In this work, we carried out calculations to simulate the interactions between ultrashort laser pulse and atom. We carried out the simulation using the classical trajectory Monte Carlo (CTMC) method. We determined the effect of the GDD, TOD and Fourth Order Dispersion (FOD) on the photoelectron spectra and ionisation probabilities arising from the interactions. We performed calculations for hydrogen and krypton atoms at intensity of $6.5 \times 10^{14}~\mathrm {W/cm^2}$, 7 fs intensity FWHM, and 800 nm central wavelength. The laser pulse was linearly polarised along the *z* axis. We have also investigated how the left-right asymmetry of the photoionisation as a function of CEP and kinetic energy is influenced by the shaping of the ultrashort laser pulse with GDD. To investigate this effect we carried out calculations for Na atoms and used ultrashort pulses with intensity of $\mathrm {4 \times 10^{12}~W/cm^2}$, 4.5 fs FWHM, 800 nm central wavelength. We considered a linearly polarised pulse. This work was inspired by the results carried out by Wollenhaupt et al.^14^, Krug et al.^13^ and Zille et al.^19^. In the first two papers, the photoionised electron spectra were studied theoretically and experimentally induced by laser pulses having shaped with different GDDs. It should be noted, however, that in these papers the time-dependent Schrödinger equation was solved for three states for potassium and five states for sodium. In the work of Zille et al.^19^ the left-right asymmetry was investigated for the ATI both experimentally and theoretically. The calculations incorporated quantum effects, therefore, we did not expect perfect correspondence with our calculations and the results of Zille et al. However, we intended to grasp and qualitatively understand the basic effects of the dispersion on the electron spectra and also its influence on CEP dependence of the photoemission. The usefulness of calculations based on classical approach is demonstrated, for example by Hack et al.^20^. In the aforementioned study the ionization of hydrogen atom was investigated with quantum approaches and found that the ionized electrons can be described with classical trajectories calculated with initial conditions obtained from the quantum description of the system.

Unless otherwise stated, we used the Hartree atomic units for our calculations and in our article.

**Figure 1:**
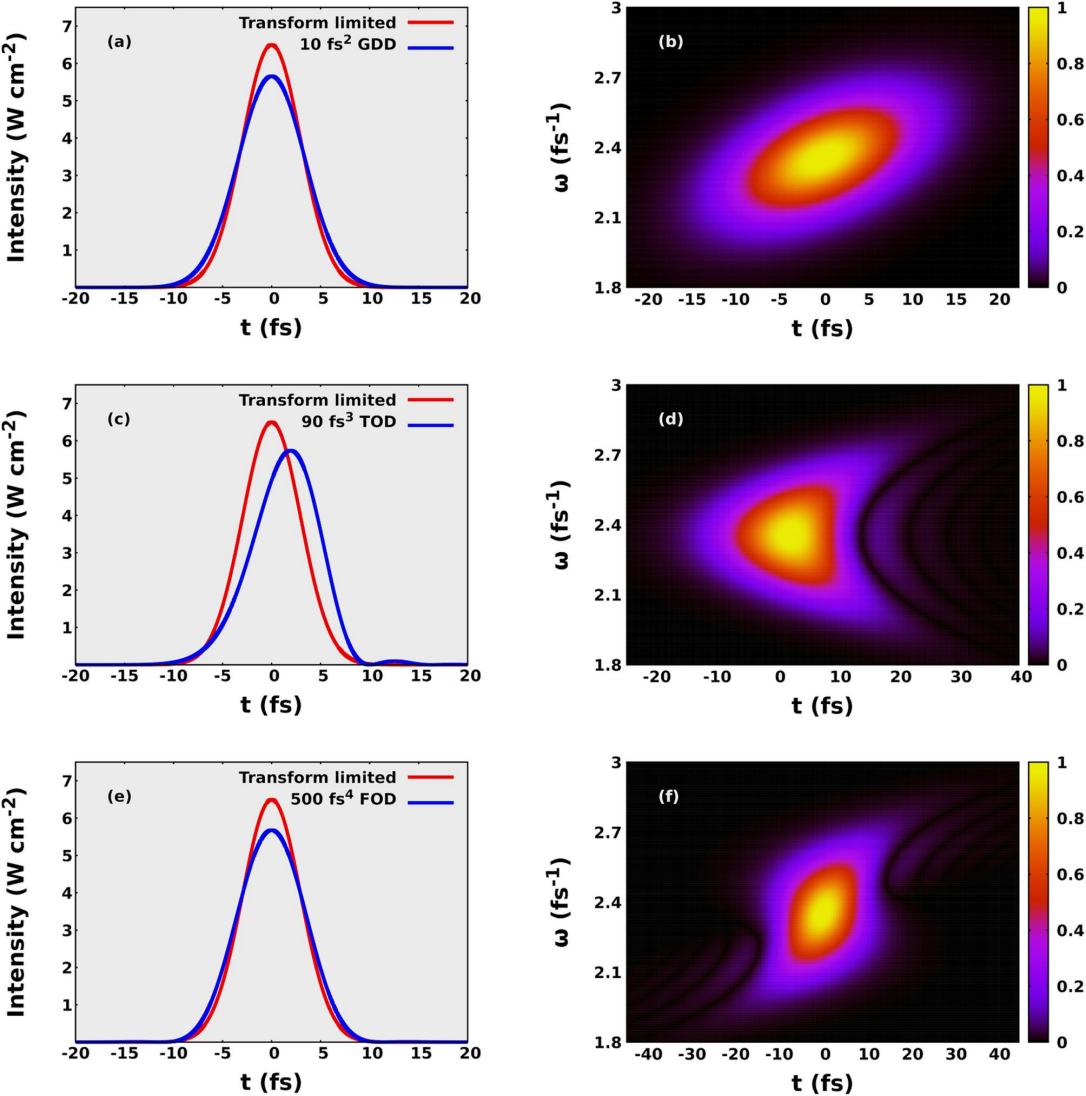
The effect of GDD, TOD and FOD on an ultrashort pulse of 7 fs FWHM and a carrier frequency of 800 nm. On the left side the intensity as a function of time is depicted. The curves denote the shape of the pulses. The red lines show the TL pulses and the blue curves denote pulses shaped with 10 fs$\mathrm {^2}$ GDD (a), 90 fs$\mathrm {^3}$ TOD (c), and 500 fs$\mathrm {^4}$ FOD (e). In the right column the corresponding Wigner distributions are plotted for 10 fs$\mathrm {^2}$ GDD (b), 90 fs$\mathrm {^3}$ TOD (d) and 500 fs$\mathrm {^4}$ FOD (f).

## Calculations

### The theory of dispersion

It is well known that the dispersion means the dependence of the phase velocity on the frequency^1,21–24^. When an ultrashort laser pulse is propagating through a dispersive material it acquires spectral phase $\phi (\omega )$ resulting in the modification of the shape and frequency distribution of the pulse. In order to describe the dispersion we need to consider an ultrafast laser pulse polarized along the *z* axis in time domain as

1 $$\begin{aligned} E_{in{,z}}(t) = f(t) \exp {(i\omega _{0}t + i\varphi _{CEP})} \end{aligned}$$

where $\omega _{0}$ is the central or carrier frequency, *f*(*t*) is the envelope of the electric field and $\varphi _{CEP}$ is the CEP of the laser pulse, respectively. In the case of our calculations we considered the pulse as transform limited (TL), and Gaussian shape was assumed for the temporal intensity envelope. Therefore, the envelope of the electric field can be described by the expression

2 $$\begin{aligned} f(t) = E_0 \sqrt{\exp \left( -4\ln (2)\frac{t^2}{\tau _0^2}\right) }, \end{aligned}$$

where the $E_0$ is the amplitude and $\tau _0$ is the intensity FWHM of the pulse. The pulse in frequency domain can be obtained as the Fourier transform of the pulse in time domain

3 $$\begin{aligned} {\tilde{E}}_{in{,z}}(\omega ) = \frac{1}{\sqrt{2\pi }}\int _{-\infty }^{\infty } E_{in{,z}}(t)\exp (-i\omega t) dt. \end{aligned}$$

When an ultrashort laser pulse is propagating in a dispersive medium, the dispersion can be described by the spectral phase $\phi (\omega ) = \beta (\omega ) L$, where $\beta (\omega )$ is the propagation constant and *L* is the thickness of the dispersive medium. After propagation the laser pulse can be written in the form

4 $$\begin{aligned} {\tilde{E}}_{out{,z}}(\omega ) = {\tilde{E}}_{in{,z}}(\omega )\exp (-i\phi (\omega )), \end{aligned}$$

where both the propagation constant and the spectral phase can be written in a Taylor series

5 $$\begin{aligned} \phi (\omega ) = L \sum _n \frac{1}{n!} \frac{d^n\beta }{d\omega ^n}\Bigg \vert _{\omega =\omega _0}(\omega - \omega _0)^n = \sum _n \frac{1}{n!} \frac{d^n\phi }{d\omega ^n}\Bigg \vert _{\omega =\omega _0}(\omega - \omega _0)^n \end{aligned}$$

where the *n*-th derivative means the *n*-th order dispersion described in units of fs$\mathrm {^n}$. Here the 0-th order means the change of the CEP phase of an ultrashort pulse during its propagation in dispersive medium. As we took into account the CEP of the incoming laser pulse we did not consider its change in medium. The first derivative is the Group Delay (GD) which is the group velocity showing how fast the laser pulse propagates in a given medium. As we performed our calculations with laser pulses already left the dispersive medium and interacts with atoms in vacuum, we obviously did not consider the GD in our calculations. The GDD leads to temporal broadening of the pulse and the variation of frequency as a function of time. This variation is called the chirp of the laser pulse, and GDD causes linear chirp. The shaping of an ultrashort laser pulse with TOD entails the appearance of pre- or post-pulses depending on the sign of the TOD. This means that the temporal profile of a pulse shaped with TOD is temporally asymmetric. The FOD modifies the temporal profile of the pulse and also broadens it. It also modifies the temporal variation of the frequency, although it is not linear in nature. In this article we report our calculations performed with GDD, TOD and FOD defined by the expressions below:

6 $$\begin{aligned} GDD = \frac{1}{2} \frac{d^2\phi }{d\omega ^2}\Bigg \vert _{\omega =\omega _0}\end{aligned}$$

7 $$\begin{aligned} TOD = \frac{1}{6} \frac{d^3\phi }{d\omega ^3}\Bigg \vert _{\omega =\omega _0}\end{aligned}$$

8 $$\begin{aligned} FOD = \frac{1}{24} \frac{d^4\phi }{d\omega ^4}\Bigg \vert _{\omega =\omega _0} \end{aligned}$$

This means that in Eq. (4) the function $\phi (\omega )$ was calculated applying the approximations $\phi ^{(2)}=GDD\, (\omega - \omega _{0})^{2}$, $\phi ^{(3)}=TOD\, (\omega - \omega _{0})^{3}$, and $\phi ^{(4)}=FOD\, (\omega - \omega _{0})^{4}$. The complex output pulse in time domain can be obtained with the inverse Fourier transformation of ${\tilde{E}}_{{out,z}}(\omega )$

9 $$\begin{aligned} E_{out{,z}}(t) = \frac{1}{\sqrt{2\pi }}\int _{-\infty }^{\infty }{\tilde{E}}_{out,z}(\omega )\exp (i\omega t) d\omega . \end{aligned}$$

In order to represent an ultrashort pulse in time and frequency domain, and to visualize the dispersion as well, we need to use Wigner function^25–28^. The latter function was originally introduced by Wigner to represent the quantum states in phase space^29^. The representation was later proved to be useful in signal analysis^30,31^ and for ultrashort light pulses as well^32,33^. The Wigner function $W(t, \omega )$ in the latter case is expressed as

10 $$\begin{aligned} W(t, \omega ) = \frac{1}{\sqrt{2\pi }} \int _{-\infty }^{\infty } E\left( t + \frac{\tau }{2}\right) E^* \left( t-\frac{\tau }{2}\right) \exp (-i\omega \tau ) d\tau \end{aligned}$$

11 $$\begin{aligned} W(t, \omega ) = \frac{1}{\sqrt{2\pi }} \int _{-\infty }^{\infty } \tilde{E}\left( \omega + \frac{\Omega }{2}\right) \tilde{E}^*\left( \omega -\frac{\Omega }{2}\right) \exp (i\Omega t) d\Omega \end{aligned}$$

where ${\tilde{E}}(\omega )$ and *E*(*t*) are the *z* component of the complex electric field in the frequency and time domain, respectively. The temporal intensity envelope and the Wigner representation of ultrashort pulses shaped with GDD, TOD and FOD with the corresponding TL pulses are depicted in Fig. 1.

### The classical trajectory monte carlo method

For our calculations we used the CTMC method originally introduced by Abrines and Percival to simulate ion collisions with H atoms^34^. Later CTMC was generalised for multielectron atoms^35^, and was proved to be a useful method to describe ion-atom collisions^36–40^. Although numerous works dealing with soft-Coulomb potential^41–48^, as it was pointed out by Sarkadi^49^ the screened potential^50^ is in better agreement with the results obtained by quantum mechanical calculations. In the present work the applied screened potential for the electron-core interaction was chosen as

12 $$\begin{aligned} V(r) = \frac{f_{scr}(r)}{r} \end{aligned}$$

where $f_{scr}(r)$ is written as

13 $$\begin{aligned} f_{scr}(r) = Z-(N-1)\left[ 1 - \frac{1}{(e^{r\xi } - 1)\eta /\xi + 1}\right] . \end{aligned}$$

Here *Z* means the nuclear charge of the target, *N* is the number of electrons in the target atom^51^. The $\eta$ and $\xi$ are parameters determined by Garvey et al.^50^ using the technique that minimizes the total energy of the atom^52^. The $\eta$ and $\xi$ vales are 4.418 and 1.351 for Kr, and 2.85 and 1.712 for Na respectively. The CTMC considers the atom as an object consisting of an electron and the atomic nucleus with the rest of the electrons, i.e. it is a one-active electron approach. For the simulation of ion-atom collisions the whole process is a three-body problem where the classical Newtons equations are solved under different initial conditions according to the prescription given in Ref.^35^. The CTMC was later developed to be able to simulate the atomic interactions with ultrashort pulses^53–64^. In our calculations we treated the process as a two-body problem in the outer electric field of the ultrashort pulse and the equations of motions were solved with the Runge-Kutta Dormand-Prince method^65^. Therefore the equations of motion for the electron and the target are

14 $$\begin{aligned} m_e \ddot{\mathbf {r}}_{e} = \frac{Z_eZ_t (\mathbf {r}_{e} - \mathbf {r}_{t})}{|\mathbf {r}_{e} - \mathbf {r}_{t}|^2}\left[ \frac{f_{scr}(|\mathbf {r}_{e} - \mathbf {r}_{t}|)}{|\mathbf {r}_{e} - \mathbf {r}_{t}|} - f^{'}_{scr}(|\mathbf {r}_{e} - \mathbf {r}_{t}|)\right] + Z_e \mathbf {E}_e(t) \end{aligned}$$

15 $$\begin{aligned} m_e \ddot{\mathbf {r}}_{t} = \frac{Z_eZ_t (\mathbf {r}_{t} - \mathbf {r}_{e})}{|\mathbf {r}_{t} - \mathbf {r}_{e}|^2}\left[ \frac{f_{scr}(|\mathbf {r}_{t} - \mathbf {r}_{e}|)}{|\mathbf {r}_{t} - \mathbf {r}_{e}|} - f^{'}_{scr}(|\mathbf {r}_{t} - \mathbf {r}_{e}|)\right] + Z_t \mathbf {E}_t(t) \end{aligned}$$

where $m_e$ and $m_t$ are the mass, $\mathbf {r}_{e}$, $\mathbf {r}_{t}$ are the position and $Z_e$ and $Z_t$ are the charge of the electron and target respectively. $\mathbf {E}_e(t)$ and $\mathbf {E}_t(t)$ are the electric field at the position of the electron and the target, and

16 $$\begin{aligned} f^{'}_{scr}(r) = \frac{\eta (1 - N) e^{r\xi }}{\left[ \left( e^{r\xi } - 1 \right) \eta /\xi + 1 \right] ^2}. \end{aligned}$$

**Figure 2:**
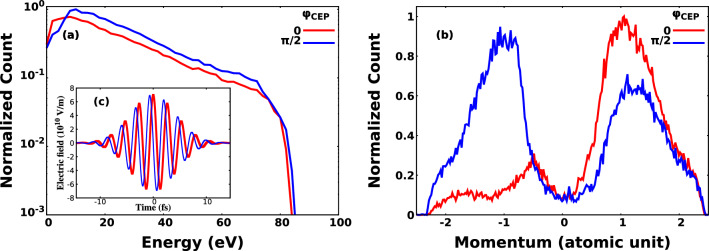
Photoelectron spectra (**a**) and momentum distributions (**b**) at $\varphi _{CEP} = 0$ and $\pi /2$. In the inset (**c**) the time dependence of the electric field strength at $\varphi _{CEP} = 0$ and $\pi /2$ is depicted.

The CEP of the few cycle laser pulses may influence the properties of the electron spectra^15–17^. We employed a laser pulse of 7 fs FWHM. The effect of CEP on the calculated electron spectrum is clearly seen in Fig. 2: the CEP influences the calculated electron spectrum. As we intended to investigate the effect of the *n*-th order dispersion on electron spectra, we averaged the results of the calculations over CEP. To do this, we determined the waveform of the electric field for 120 different CEP angles ranging from 0$\mathrm {^{\circ }}$ to 357$\mathrm {^{\circ }}$ with a step of 3$\mathrm {^{\circ }}$ in between them for each dispersion parameters. We calculated 24000 and 120000 trajectories for krypton and for hydrogen, respectively, for the different CEP values, and we summed up the results of the trajectories. For the study of the effect of the dispersion on the left-right asymmetry of the photoelectron spectra for sodium we performed our calculations with 60 different CEP values ranging from 0$\mathrm {^\circ }$ to 354$\mathrm {^{\circ }}$ with a step of 6$\mathrm {^{\circ }}$ in between them. For a given CEP value we calculated 1.2 million trajectories, and we performed our calculations for five different GDD values.

## Results

**Figure 3:**
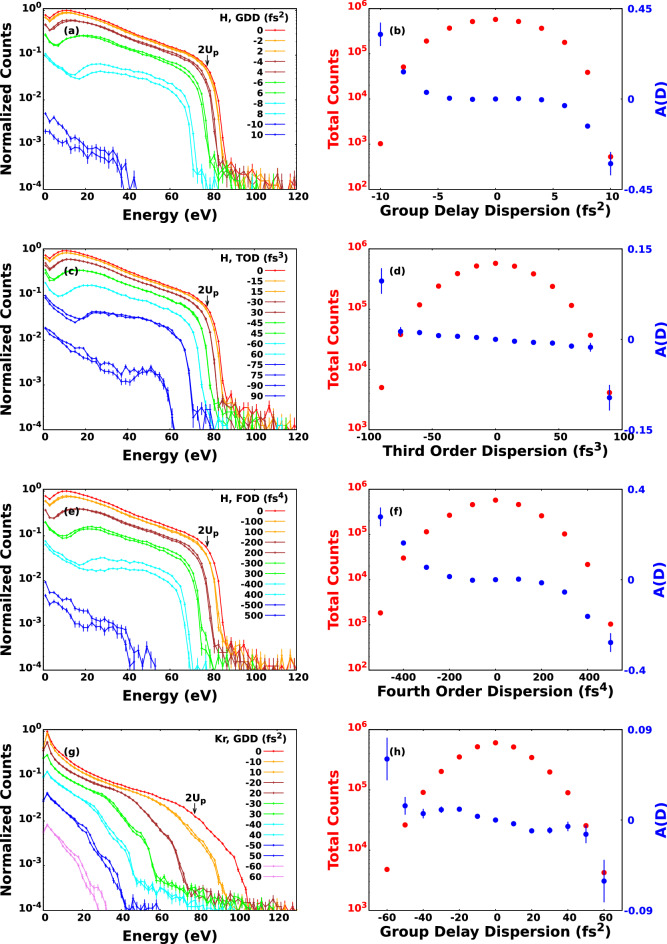
Photoelectron spectra for hydrogen calculated with different GDD (**a**), TOD (**c**) and FOD (**e**). The corresponding number of electron counts and asymmetry parameters defined by Eq. (17) can be seen on the right column with error bars. Panels (**g** and **h**) refer for krypton and only for GDD. On the left column we have marked $2U_p$ where $U_p$ means the ponderomotive potential at $\mathrm {6.5 \times 10^{14} W/cm^2}$ intensity.

**Figure 4:**
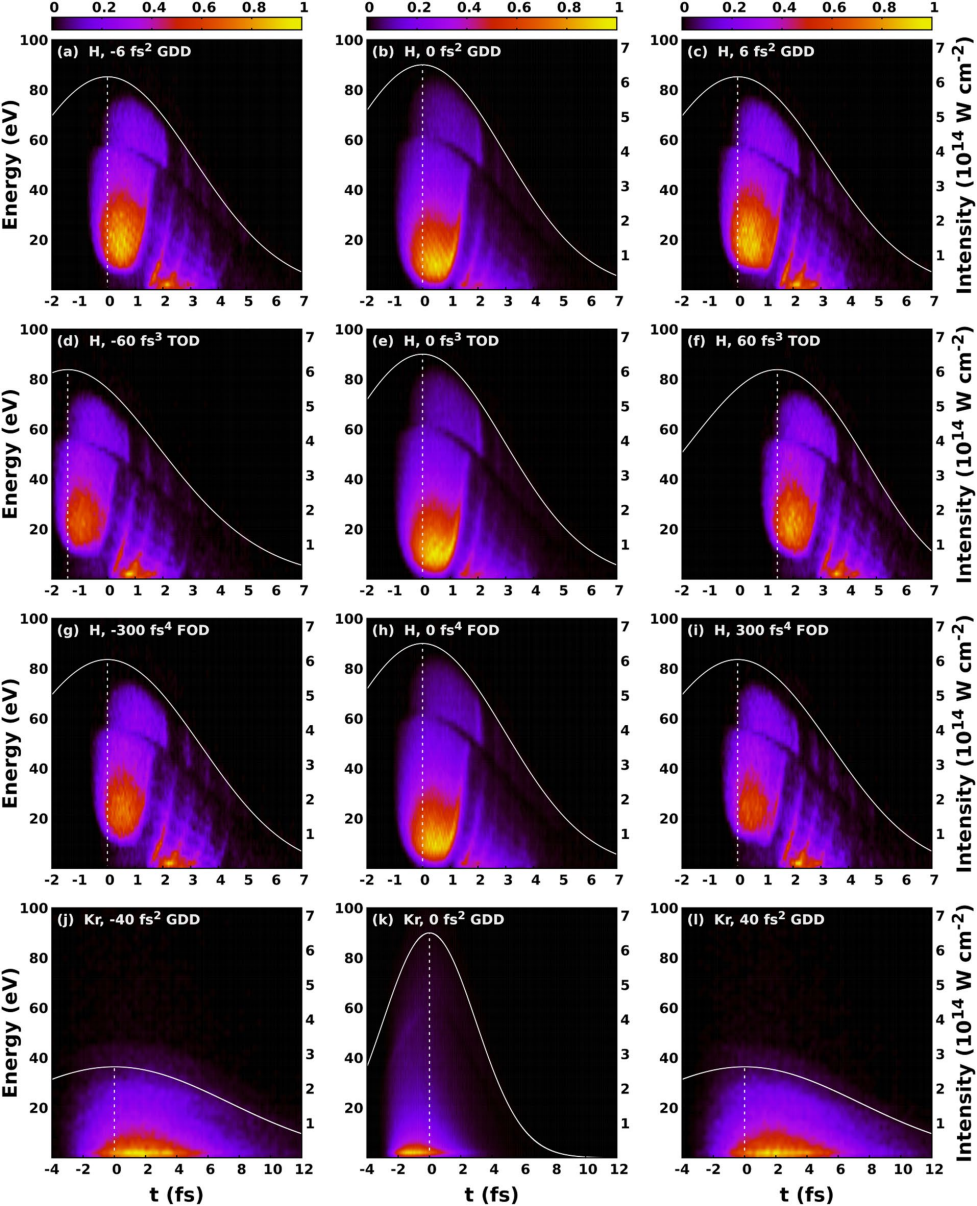
Contour plots of the normalized photoelectron distributions as a function of ionisation time and kinetic energy. The temporal envelope of the intensity of the laser pulse is depicted with a white line. The vertical dashed white line shows the moment of time when the intensity is maximal. Photoelectron distributions for hydrogen calculated with different GDD (**a–c**), TOD (**d–f**) and FOD (**g–i**). Photoelectron distributions for krypton calculated with different GDD (**j–l**).

**Figure 5:**
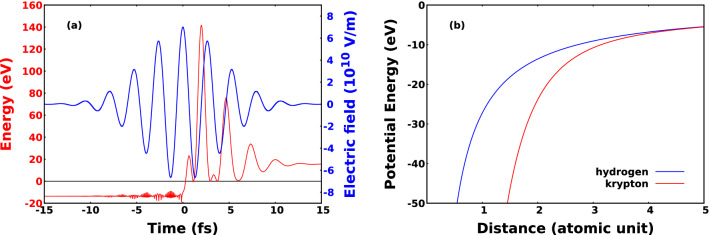
Electron energy and incident electric field of the ultrashort laser pulse as a function of time (**a**). The applied laser intensity is $\mathrm {6.5 \times 10^{14} W/cm^2}$. The time of ionisation is the moment when the energy of electron reaches 0 eV at the first time. Atomic potentials of hydrogen and krypton atoms (**b**) defined in Eqs. (12)-(13).

**Figure 6:**
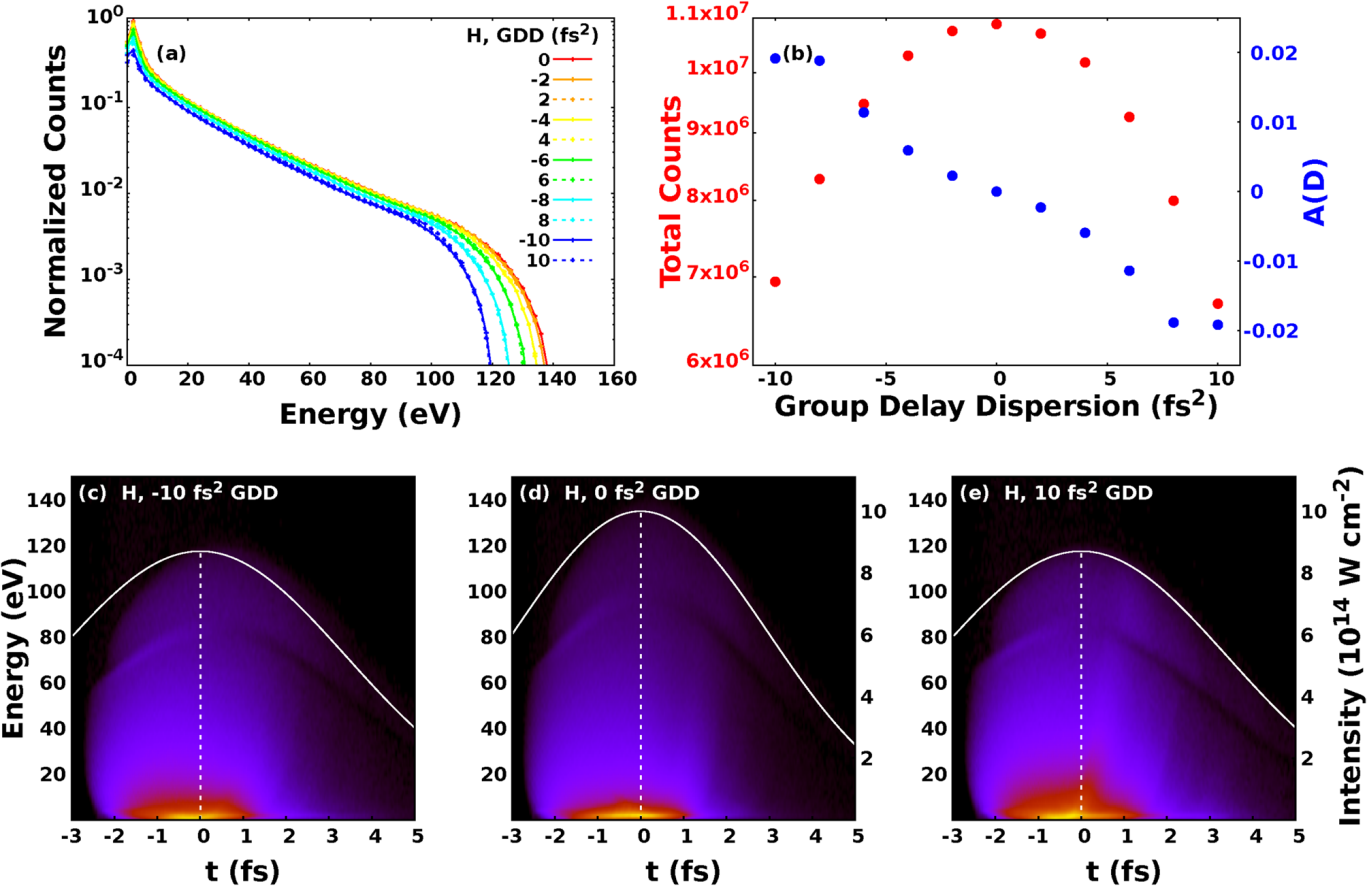
Calculated photoelectron spectra for hydrogen at $\mathrm {10^{15} W/cm^2}$ intensity with different values of GDD (**a**) and the corresponding electron yield and asymmetry parameter (**b**) defined by Eq. (17). Contour plots of the square root of the normalized photoelectron distributions as a function of ionisation time and kinetic energy with -10 fs$\mathrm {^2}$ (**c**), 0 fs$\mathrm {^2}$ (**d**) and 10 fs$\mathrm {^2}$ (**e**) GDD. The temporal envelope of the intensity of the laser pulse is depicted with a white line. The vertical dashed white line shows the moment of time when the intensity is maximal.

In order to quantitatively characterize the effect caused by the *n*-th order dispersion (D) in the electron spectra we introduced the asymmetry parameter as

17 $$\begin{aligned} A(D) = \frac{N\left( D\right) - N\left( -D\right) }{N\left( D\right) + N\left( -D\right) } \end{aligned}$$

where *N*(*D*) is the number of photoelectrons at a given value of dispersion. In Fig. 3 the calculated electron spectra with the corresponding electron yield and asymmetry parameters are plotted. Both the electron spectra and the electron yield are summed for all the calculated CEP value. The asymmetry parameters clearly show the effect of the dispersion on the number of the electrons. Although this asymmetry is present in GDD, TOD, and FOD as well, it is slightly less pronounced for TOD. According to the electron spectra depicted in Fig. 3 the electron yield decreases with increasing absolute value of GDD, TOD, and FOD as well. This feature can be attributed to the fact that the intensity of the pulse shaped with GDD, TOD or FOD is always smaller than the TL one as it can be seen in Fig. 1. This smaller amplitude will result in smaller electron yields and cutoff energies. For photoelectron spectra induced with linearly polarised laser pulse, the cutoff energy is approximately $\mathrm {2U_p}$, where $\mathrm {U_p}$ is the ponderomotive potential^66^. The ponderomotive potential is expressed as^67^ $U_p = e^2E_0^2/4m\omega ^2$, where $\omega$ is the angular frequency of the incoming light, $m_e$ and *e* mean the mass and electric charge of the electron, respectively, and $E_0$ is the amplitude of the electric field. The electron spectra induced with negative GDD or FOD have bigger cutoff values compared to those induced with the same amount of GDD or FOD in absolute value but having positive sign. Interestingly this effect is not relevant or at least can not be seen for TOD. In order to better understand the physical origin of these dispersion dependent effects, in Fig. 4 we have plotted the distribution of electrons as a function of ionisation time and kinetic energy for hydrogen and for krypton as well for various values of GDD, TOD, and FOD. The incident intensity can also be seen in the figure. We can conclude that most electrons are ionised after the moment of time when the incident intensities are maximal. This feature can be attributed to the fact that in the classical picture the incident laser pulse modifies the energy of the bound electron, and in case of ionization the electron accumulates energy gradually as it can be seen in Fig. 5a. At moderately low intensities most electrons can gain enough energy to be ionised after the moment of time when the incident intensity is maximal. Consequently the incident electric field has a different angular frequency and thereby ponderomotive potential at the time of ionisation, depending on the sign of the chirp when the pulse is shaped with GDD and FOD. According to the form of the ponderomotive potential (see above), the ionisation is more probable when the pulse is down chirped, namely when the incident frequency decreases with time. This leads to higher ionisation rates and electron energies for down chirped pulses, and the effect is more pronounced for pulses shaped with the higher GDD and FOD in absolute value but having opposite sign. These observations are completely in agreement with the results presented in Fig. 3. The dispersion dependent effect is also present for TOD, although it is not pronounced as much as it is for pulses shaped with GDD and FOD. To understand this better, we need to consider the Wigner function of the pulse shaped with TOD in Fig. 1d and compare them with those corresponding to pulses shaped with GDD and FOD in Fig. 1b and f. As far as GDD and FOD are concerned we can observe monotonity of frequency as a function of time in sharp contrast to the TOD where both the low and high frequency parts are delayed with the same amount of time. The above arguments can be tested performing calculations at higher intensities. When the intensity is high enough we can not state that most electrons are ionized after the moment when the incident intensity is maximal. The frequency and ponderomotive potential at the time of ionisation are random in nature, and not or much less depend on the chirp if we consider the whole ensemble. In Fig. 6 the results of calculations carried out for hydrogen at $\mathrm {10^{15} W/cm^2}$ intensity are displayed. We can observe from Fig. 6c–e that most of the ionisation does not occur after the moment when the incident intensity is maximal. As it can be seen in Fig. 6a and b, the asymmetric nature of the photoionisation process is still present, but the effect is much less pronounced compared to the results obtained at lower intensities: The change of the asymmetry parameter is smaller by more than one order of magnitude. We must pay attention to the peculiar structure of the ionisation time - energy distributions of the electron emission from hydrogen atom depicted in Fig. 4a–i. The vast majority of electrons belong to three visibly distinguishable groups, namely, electrons with the highest kinetic energies, the electrons with moderately high energies and low-energy electrons ionised with some delay. This third groups of electrons display a periodic, oscillating structure that cannot be explained with the wavelength of the incident pulse as the period of oscillations are typically lower than 1 fs meanwhile the period of a pulse with a central wavelength of 800 nm is 2.67 fs. One might attribute this feature to the incompleteness of our calculations, namely the relatively small number of different CEP values with which we performed our calculations. To check this, we carried out calculations with TL pulses at the same wavelength, intensity, and pulse width but the CEP values were random numbers. We found that the results were exactly the same what we obtained with calculations with 120 fixed CEP values. We call the attention to the difference of the ionisation probabilities observed between the hydrogen and krypton atoms. The ionisation was significantly more probable for krypton than for hydrogen in spite of the fact that the outer electron of the krypton atom is slightly more bounded than that of the hydrogen. This effect can probably be attributed to the difference of their atomic potentials defined by Eqs. (12), (13). and depicted in Fig. 5b.

**Figure 7:**
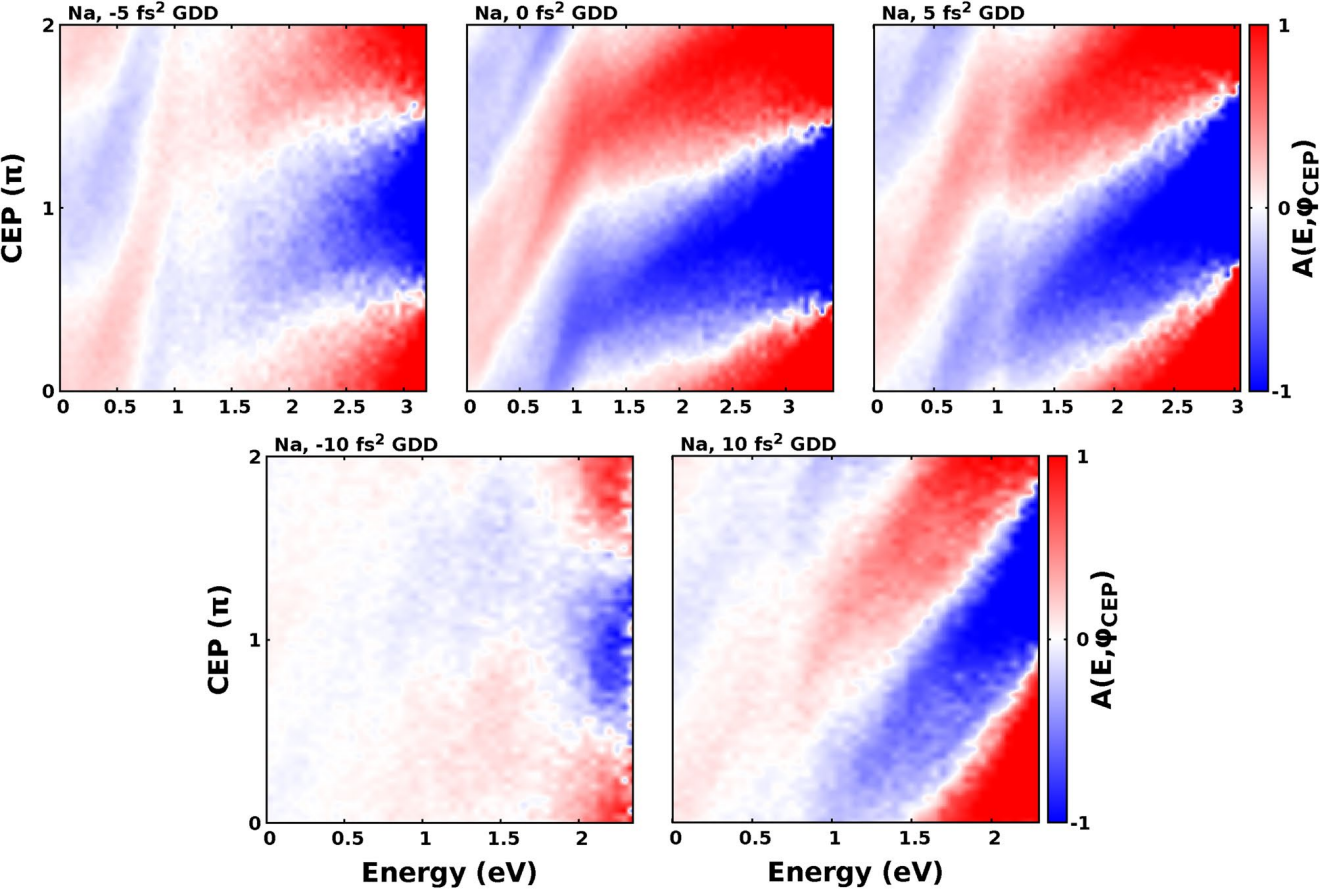
Calculated maps of the asymmetry defined by Eq. (18) for Na at $\mathrm {4 \times 10^{12} W/cm^2}$ intensity with different values of GDD.

**Figure 8:**
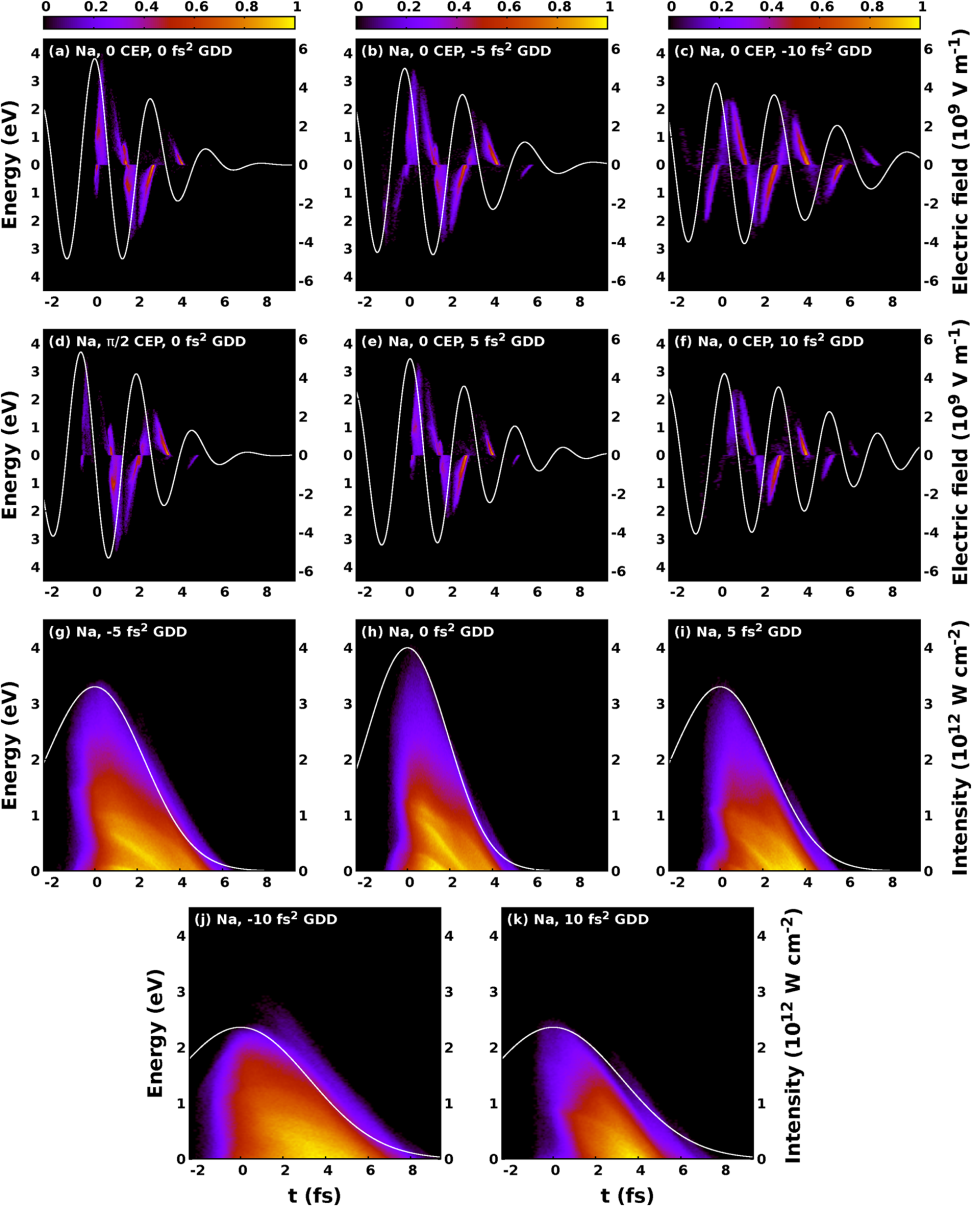
Contour plots of the square root of the normalized photoelectron distributions as a function of ionisation time and kinetic energy for different GDD and CEP values (**a–f**). The temporal profile of the electric field of the laser pulse is depicted with a white line. The energy axis is divided into an upper and lower panel representing electrons with positive and negative final momenta along the polarization axis. Panels (**g–k**) display contour plots of the square root of the normalized photoelectron distributions as a function of ionisation time and kinetic energy for different GDD values. The photoelectron distributions are CEP summed. The temporal envelope of the intensity of the laser pulse is depicted with a white line.

In the followings, we discuss how the GDD influences the left-right asymmetry of the electron spectrum as a function of kinetic energy and CEP. In order to quantitatively describe the left-right asymmetry of photoelectron spectrum we introduce the asymmetry parameter as

18 $$\begin{aligned} A(E, \varphi _{CEP}) = \frac{N^+\left( E, \varphi _{CEP}\right) - N^-\left( E, \varphi _{CEP}\right) }{N^+\left( E, \varphi _{CEP}\right) + N^-\left( E, \varphi _{CEP}\right) } \end{aligned}$$

where $N^+$ and $N^-$ mean the number of electrons having positive and negative momentum along the polarization axis respectively. The calculated $A(E, \varphi _{CEP})$ asymmetry maps are plotted in Fig. 7 for sodium atoms. The applied laser intensity was $4 \times 10^{12} \mathrm {W/cm^2}$. We performed the calculations for the TL case and for pulses shaped with $\pm 5~\mathrm {fs^2}$ and $\pm ~10~\mathrm {fs^2}$ respectively. The FWHM of the TL pulse was 4.5 fs, meanwhile the FWHM of the pulses shaped with $\pm 5~\mathrm {fs^2}$ and $\mathrm {\pm ~10~fs^2}$ GDD were $5.45~\mathrm {fs}$ and $7.62~\mathrm {fs}$ respectively. Kübel et al.^68^ studied the $A(E, \varphi _{CEP})$ asymmetry map for the ATI process for caesium atoms experimentally and theoretically as well. Zhou et al.^18^ carried out a similar investigation for xenon atoms. The asymmetry maps obtained by the latter authors can be found in Fig. 2a–c in Ref.^18^. They made their experiment at $1.1 \times 10^{14} \mathrm {W/cm^{2}}$ intensity, 780 nm central wavelength, and 5 fs pulse width. Although they performed their studies with parameters differing from those of the present investigations, especially as far as the applied laser intensity and studied element are concerned, we can observe a similarity between their and our results. We also note that their asymmetry maps cover a considerably higher electron energy range compared to ours. However, we did not expect a perfect correspondence between their studies and ours due to the different theoretical approaches. We can conclude from Fig. 7 that with increasing GDD in absolute value the effect of CEP on the photoelectron spectrum decreases. This phenomenon can be attributed to the broadening of the pulse as a result of the shaping. This broadening results in the increased number of the optical cycles. As the number of optical cycles increases the CEP dependent effects will be less and less pronounced. The CEP dependence of the electron spectra induced with pulses shaped with positive GDD is more pronounced compared to those spectra induced with pulses shaped with the same amount of GDD in absolute value but having negative sign. This phenomenon can be attributed to the difference of the time interval, namely the time window when the electrons are ionised. We can easily observe from Fig. 8 and especially from Fig. 8g–k that the time window increases with the increasing GDD, but it is considerably shorter for electron spectra obtained by pulses shaped with positive GDD compared to the case when it is obtained with the same amount of GDD in absolute value but with negative sign. This explains the phenomenon why the most pronounced CEP dependence can be observed with electron spectra induced with TL pulse, and why the CEP dependence is more pronounced with electron spectra induced with ultrashort pulses shaped with positive GDD. In order to better understand the physical origin of the chirp dependence of the time window we need to consider the distribution of electrons as a function of ionization time and kinetic energy. Most electrons are ionised after the moment when the incident intensity is maximal, as it can be seen in Fig. 8. Positively chirped pulses has lower ponderomotive potential after the moment when the incident intensity is maximal compared to pulses with negative chirp. This explains the shorter period of time windows with positively chirped pulses comparing to pulses with negative chirp. In Fig. 8 the contour plots depicting the square root of the electron distributions can be seen as a function of time and energy. In panels Fig. 8a–f the corresponding electric field, while in panels Fig. 8g–k the corresponding intensity is depicted with the white line. As it can be seen in Fig. 8a–f, the ionisation process is determined by the temporal profile of the electric field, and the ionisation time influences the final energy of the electron. The later an electron is ionised, the less the final energy that it can acquire, namely the electrons with high kinetic energies are ionised at the beginning of the time window. We can also conclude from Fig. 8a–f the validity of the simpleman model^66^ in our calculations. According to this model the velocity of the electron is $\mathbf {v} = \mathbf {v}(t_0) + e/m\int _{t_0}^{t} \mathbf {E}(t') dt'$, i.e. one expects about $\mathrm {\pi /2}$ phase difference between the kinetic energy and the electric field. Considering the form of the applied electric field described in Eq. (9) we can conclude that the simpleman model is in good agreement with our calculations.

## Conclusion

In this paper, we presented results of classical calculations for the energy distributions and yield for hydrogen and krypton atoms using ultrafast laser pulses having shaped with different second- (GDD), third- (TOD) and fourth- (FOD) order dispersions. We have also studied how the CEP dependent effects and ultimately the left-right asymmetry of an electron spectrum are influenced in case of pulses having different GDDs. For the investigation of the energy distributions and yield for hydrogen and krypton, the width of the applied TL pulse was 7 fs while for the left-right asymmetry calculations it was 4.5 fs. The central frequency was 800 nm in both cases. We found that the electron yield and CEP effects depend not only on the absolute value but the sign of the dispersion as well. For negative GDD, TOD and FOD we obtained a higher number of electron counts. In the case of GDD and FOD, we also observed this asymmetry for the cutoff energies. We also found that the CEP effects decrease with increasing amount of GDD in absolute value. The same but more pronounced tendency was observed for pulses shaped with negative GDD.

## References

[1] Walmsley I, Waxer L, Dorrer C (2001). The role of dispersion in ultrafast optics. Rev. Sci. Instrum..

[2] Strickland D, Mourou G (1985). Compression of amplified chirped optical pulses. Opt. Commun..

[3] Danson CN (2019). Petawatt and exawatt class lasers worldwide. High Power Laser Sci. Eng..

[4] Dantus M, Lozovoy VV (2004). Experimental coherent laser control of physicochemical processes. Chem. Rev..

[5] Assion A, Baumert T, Helbing J, Seyfried V, Gerber G (1996). Coherent control by a single phase shaped femtosecond laser pulse. Chem. Phys. Lett..

[6] Kaufman B, Rozgonyi T, Marquetand P, Weinacht T (2021). Competition between dynamic resonance and internal conversion in strong-field molecular ionization with chirped ultrafast laser pulses. Phys. Rev. A.

[7] Reusch N, Griebe S, Karges J, Weitzel KM (2015). Photoionization Yields in Intense fs-Laser Fields - A Systematic Investigation of Chirp Effects. Zeitschrift fur Physikalische Chemie.

[8] Natan A (2012). Quantum control of photodissociation by manipulation of bond softening. Phys. Rev. A.

[9] Lev U (2015). Quantum control of photodissociation using intense, femtosecond pulses shaped with third order dispersion. J. Phys. B: Atomic, Mol. Opt. Phys..

[10] Reusch N, Krein V, Wollscheid N, Weitzel KM (2018). Distinction of structural isomers of benzenediamin and difluorobenzene by means of chirped femtosecond laser ionization mass spectrometry. Zeitschrift fur Physikalische Chemie.

[11] Schäfer V, Weitzel K-M (2020). Qualitative and quantitative distinction of ortho-, meta-, and para-fluorotoluene by means of chirped femtosecond laser ionization. Anal. Chem..

[12] Nakajima T (2007). Above-threshold ionization by chirped laser pulses. Phys. Rev. A.

[13] Krug M (2009). Coherent strong-field control of multiple states by a single chirped femtosecond laser pulse. New J. Phys..

[14] Wollenhaupt M, Präkelt A, Sarpe-Tudoran C, Liese D, Baumert T (2006). Quantum control by selective population of dressed states using intense chirped femtosecond laser pulses. Appl. Phys. B: Lasers Opt..

[15] Apolonski A (2004). Observation of light-phase-sensitive photoemission from a metal. Phys. Rev. Lett..

[16] Földi P, Márton I, Német N, Ayadi V, Dombi P (2015). Few-cycle plasmon oscillations controlling photoemission from metal nanoparticles. Appl. Phys. Lett..

[17] Bergues B (2011). Sub-cycle electron control in the photoionization of xenon using a few-cycle laser pulse in the mid-infrared. New J. Phys..

[18] Zhou Y (2021). Carrier-envelope phase dependence of high-order above-threshold ionization by few-cycle laser pulses. J. Phys. B: Atomic, Mol. Opt. Phys..

[19] Zille D, Adolph D, Möller M, Sayler AM, Paulus GG (2018). Chirp and carrier-envelope-phase effects in the multiphoton regime: Measurements and analytical modeling of strong-field ionization of sodium. New J. Phys..

[20] Hack S, Majorosi S, Benedict MG, Varró S, Czirják A (2021). Quantum interference in strong-field ionization by a linearly polarized laser pulse and its relevance to tunnel exit time and momentum. Phys. Rev. A.

[21] Weiner AM (2009). Ultrafast Optics.

[22] Weiner AM (2000). Femtosecond pulse shaping using spatial light modulators. Rev. Sci. Instrum..

[23] Börzsönyi A, Kovács AP, Osvay K (2013). What we can learn about ultrashort pulses by linear optical methods. Appl. Sci..

[24] Präkelt A (2003). Compact, robust, and flexible setup for femtosecond pulse shaping. Rev. Sci. Instrum..

[25] Alonso MA (2011). Wigner functions in optics: Describing beams as ray bundles and pulses as particle ensembles. Adv. Opt. Photon..

[26] Walmsley IA, Dorrer C (2009). Characterization of ultrashort electromagnetic pulses. Adv. Opt. Photon..

[27] Yeremenko S, Baltuška A, Pshenichnikov MS, Wiersma DA (2000). The criterion of pulse reconstruction quality based on Wigner representation. Appl. Phys. B.

[28] Dorrer C, Walmsley IA (2005). Concepts for the temporal characterization of short optical pulses. EURASIP J. Adv. Signal Process..

[29] Wigner E (1932). On the quantum correction for thermodynamic equilibrium. Phys. Rev..

[30] Ville J (1948). Théorie et applications de la notion de signal analytique. Câbles Transm..

[31] Dragoman D (2005). Applications of the Wigner distribution function in signal processing. EURASIP J. Adv. Signal Process..

[32] Paye J (1992). The chronocyclic representation of ultrashort light pulses. IEEE J. Quantum Electron..

[33] Lerch S, Stefanov A (2016). Ultrashort pulses characterization by quantum state tomography. Opt. Express.

[34] Abrines R, Percival IC (1966). Classical theory of charge transfer and ionization of hydrogen atoms by protons. Proc. Phys. Soc..

[35] Reinhold CO, Falcón CA (1986). Classical ionization and charge-transfer cross sections for $${{\rm H}}^{+}$$ + He and $${{\rm H}}^{+}$$ + $${{\rm Li}}^{+}$$ collisions with consideration of model interactions. Phys. Rev. A.

[36] Sarkadi L (2018). Fully differential cross sections for the single ionization of helium by fast ions: Classical model calculations. Phys. Rev. A.

[37] Frémont F (2018). Transverse momentum transfer distributions following single ionization in 3.6 MeV/amu Au$${{\rm ^{53+}}}$$ + He Collisions: A 4-body classical treatment. Atoms.

[38] Wood CJ, Olson RE, Schmitt W, Moshammer R, Ullrich J (1997). Momentum spectra for single and double electron ionization of He in relativistic collisions. Phys. Rev. A.

[39] Ghavaminia H (2017). Electron capture from H$${{\rm {_2}}}{ molecule by He{{\rm {^+}}}}$$ ions. Euro. Phys. J. D.

[40] Sarkadi L (2016). Classical trajectory Monte Carlo model calculations for ionization of the uracil molecule by impact of heavy ions. J. Phys. B: Atomic, Mol. Opt. Phys..

[41] Brabec T, Ivanov MY, Corkum PB (1996). Coulomb focusing in intense field atomic processes. Phys. Rev. A.

[42] Truong TD (2022). Soft parameters in Coulomb potential of noble atoms for nonsequential double ionization: Classical ensemble model and simulations. Comput. Phys. Commun..

[43] Huang C, Zhou Y, Zhang Q, Lu P (2013). Contribution of recollision ionization to the cross-shaped structure in nonsequential double ionization. Opt. Express.

[44] Zhang Z, Zhang J, Bai L, Wang X (2015). Transition of correlated-electron emission in nonsequential double ionization of Ar atoms. Opt. Express.

[45] Liao Q (2017). Coulomb-repulsion-assisted double ionization from doubly excited states of argon. Phys. Rev. A.

[46] Zhou Y, Liao Q, Lu P (2010). Asymmetric electron energy sharing in strong-field double ionization of helium. Phys. Rev. A.

[47] Ma X, Zhou Y, Lu P (2016). Multiple recollisions in strong-field nonsequential double ionization. Phys. Rev. A.

[48] Sarkadi L (2020). Laser-induced nonsequential double ionization of helium: Classical model calculations. J. Phys. B: Atomic, Mol. Opt. Phys..

[49] Sarkadi L (2021). Comparative study of classical theoretical descriptions of the ionization of atoms induced by few-cycle laser pulses. Phys. Rev. A.

[50] Garvey RH, Jackman CH, Green AES (1975). Independent-particle-model potentials for atoms and ions with $$36 < Z\le 54$$ and a modified Thomas-Fermi atomic energy formula. Phys. Rev. A.

[51] Green AES, Sellin DL, Zachor AS (1969). Analytic independent-particle model for atoms. Phys. Rev..

[52] Bass JN, Green AE, Wood JH (1973). An analytic independent particle model for atoms: II. Modified hartree-fock calculations for atoms. Adv. Quantum Chem..

[53] Borbély S, Tőkési K, Nagy L (2008). Ionization of the hydrogen atom by intense ultrashort laser pulses. Phys. Rev. A.

[54] Botheron P, Pons B (2009). One-electron atom in a strong and short laser pulse: Comparison of classical and quantum descriptions. Phys. Rev. A.

[55] Cohen JS (2001). Reexamination of over-the-barrier and tunneling ionization of the hydrogen atom in an intense field. Phys. Rev. A.

[56] Cohen JS (2003). Effect of tunneling on ionization of Rydberg states in intense fields: Hydrogenic atoms. Phys. Rev. A.

[57] Duchateau G, Illescas C, Pons B, Cormier E, Gayet R (2000). Ionization dynamics in interactions of atoms with ultra-short and intense laser pulses. J. Phys. B: Atomic, Mol. Opt. Phys..

[58] Eckart S (2016). Nonsequential double ionization by counterrotating circularly polarized two-color laser fields. Phys. Rev. Lett..

[59] Feeler CR, Olson RE (2000). Single ionization of Ne by intense laser fields. J. Phys. B: Atomic, Mol. Opt. Phys..

[60] Hansen JP, Lu J, Madsen LB, Nilsen HM (2001). Ionization and excitation dynamics of $$\rm H (1s)$$ in short intense laser pulses. Phys. Rev. A.

[61] Hofmann C, Landsman AS, Cirelli C, Pfeiffer AN, Keller U (2013). Comparison of different approaches to the longitudinal momentum spread after tunnel ionization. J. Phys. B: Atomic, Mol. Opt. Phys..

[62] Liu C, Hatsagortsyan KZ (2010). Origin of unexpected low energy structure in photoelectron spectra induced by midinfrared strong laser fields. Phys. Rev. Lett..

[63] Mancuso CA (2016). Controlling nonsequential double ionization in two-color circularly polarized femtosecond laser fields. Phys. Rev. Lett..

[64] Pfeiffer AN (2012). Probing the longitudinal momentum spread of the electron wave packet at the tunnel exit. Phys. Rev. Lett..

[65] Dormand JR, Prince PJ (1980). A family of embedded Runge-Kutta formulae. J. Comput. Appl. Math..

[66] Becker W, Liu X, Ho PJ, Eberly JH (2012). Theories of photoelectron correlation in laser-driven multiple atomic ionization. Rev. Mod. Phys..

[67] Eichmann U, Nubbemeyer T, Rottke H, Sandner W (2009). Acceleration of neutral atoms in strong short-pulse laser fields. Nature.

[68] Kübel M (2021). High-order phase-dependent asymmetry in the above-threshold ionization plateau. Phys. Rev. Lett..

